# Draft Genome Sequences of *Chlamydiales* Bacterium STE3 and *Neochlamydia* sp. Strain AcF84, Endosymbionts of *Acanthamoeba* spp.

**DOI:** 10.1128/MRA.00220-20

**Published:** 2020-05-14

**Authors:** Stephan Köstlbacher, Stefanie Michels, Alexander Siegl, Frederik Schulz, Daryl Domman, Somchai Jongwutiwes, Chaturong Putaporntip, Matthias Horn, Astrid Collingro

**Affiliations:** a Centre for Microbiology and Environmental Systems Science, University of Vienna, Vienna, Austria; b Department of Parasitology, Faculty of Medicine, Chulalongkorn University, Bangkok, Thailand; Indiana University, Bloomington

## Abstract

*Chlamydiales* bacterium STE3 and *Neochlamydia* sp. strain AcF84 are obligate intracellular symbionts of *Acanthamoeba* spp. isolated from the biofilm of a littoral cave wall and gills from striped tiger leaf fish, respectively. We report the draft genome sequences of these two environmental chlamydiae affiliated with the family *Parachlamydiaceae*.

## ANNOUNCEMENT

Members of the *Parachlamydiaceae* are related to the well-known human and animal pathogens Chlamydia trachomatis and Chlamydia pneumoniae. *Parachlamydiaceae* show the obligate intracellular lifestyle of chlamydiae but thrive as symbionts of free-living amoebae in the environment (1, 2). The effect of these environmental chlamydiae on their amoeba hosts ranges from beneficial to adverse depending on the bacterial strain, host organism, and environmental conditions (3–5). Their analysis helps to understand better the basic biology and evolution of all chlamydiae (2, 6). Here, we provide draft genome sequences of two amoeba symbionts affiliated with the *Parachlamydiaceae*.

*Chlamydiales* bacterium STE3 resides in *Acanthamoeba* sp. strain STE3, which was isolated from the biofilm of a littoral cave wall in Hawaii. *Acanthamoeba* sp. strain AcF84, harboring *Neochlamydia* sp. strain AcF84, was obtained from gill samples of Pristolepis fasciatus (striped tiger leaf fish) in Thailand. After axenization (7), amoebae were cultivated in peptone-yeast-glucose medium at 20°C (8). Prior to symbiont DNA isolation, amoeba cells were lysed, and host DNA was digested as described previously (9). Bacterial DNA was purified using chloroform-isoamyl alcohol extraction with isopropanol precipitation (9, 10) (STE3) and the DNeasy blood and tissue kit (Qiagen) as recommended by the manufacturer (AcF84). Sequencing libraries were prepared using the Nextera XT kit (Illumina) and sequenced on an Illumina HiSeq 2000 platform. Trimming and quality control of reads were conducted with BBMap v35.43 (https://sourceforge.net/projects/bbmap/) (bbduk minlen = 50, qtrim = rl, trimq = 25, ktrim = r, k = 25, mink = 11, hdist = 1) and FastQC v0.11.4 (11). Assemblies were performed with SPAdes v3.x.0 (Table 1) (12), screened for contamination with CheckM (13), and annotated with ConsPred v1.10 and v1.21 (Table 1) (14). Default parameters were used unless noted otherwise.

**TABLE 1:** Characteristics and accession numbers of the two chlamydial symbiont genomes

Characteristic	Data for:	
	*Chlamydiales* bacterium STE3	*Neochlamydia* sp. AcF84
Assembly	SPAdes v3.1.0	SPAdes v3.5.0
Annotation	ConsPred v1.10	ConsPred v1.21
Genome coverage (×)	2,487	810
Avg read length (bp)	120	120
No. of contigs^*a*^	63	84
Contig *N*_50_ (bp)	79,518	63,140
Completeness (%)	98.28	91.38
G+C content (%)	38.72	38.02
Assembly size (bp)	2,223,901	2,503,381
No. of coding sequences^*b*^	2,009	1,970
No. of rRNAs	3	3
No. of tRNAs	37	36
GenBank accession no.	VKHO00000000	VJOT00000000

a Contigs of >800 bp.

b CDSs of >150 bp.

The draft genome sizes and detailed information for both genomes are listed in Table 1. Both genomes show hallmarks of chlamydial genomes, e.g., a reduction in genes for metabolic pathways, but encode ATP/ADP translocases, and, among other virulence factors, a type III secretion apparatus including many potential effectors.

*Chlamydiales* bacterium STE3 and the amoeba symbiont HS-T3 (15, 16) (tentatively classified as a *Thermochlamydia* sp. [17]) form a separate lineage affiliated with the *Parachlamydiaceae* in 16S rRNA-based phylogenetic trees. In contrast to strain HS-T3, STE3 seems unable to infect mammalian or insect cell lines (15, 16). Based on an analysis of groups of orthologs using OrthoFinder (18), *Chlamydiales* bacterium STE3 shares 1,838 coding DNA sequences (CDSs) (91.5%) with other chlamydiae; 81 of these are unique to STE3 and HS-T3.

*Neochlamydia* sp. AcF84 and its closest relative, *Neochlamydia* sp. EPS4, share 1,579 CDSs (80.2%), including genes of the large effector gene families NEX1 and NEX2 (19). As seen in other *Neochlamydia* genomes, AcF84 encodes many transposases and noncoding RNAs (ncRNAs), in particular, group II introns associated with reverse transcriptase/maturase proteins.

These two genome sequences will enable a better understanding of the biology and evolution of ubiquitous protist-associated chlamydiae.

### Data availability.

The draft genome sequences of the two chlamydial symbionts have been deposited in GenBank under the accession numbers VKHO00000000 and VJOT00000000. Reads are available at the SRA database under the accession numbers PRJNA492197 and PRJNA489593. The versions described in this paper are the first versions.
